# Supplementary material for: Unintended pregnancy and contraception use among African women living with HIV: Baseline analysis of the multi-country US PEPFAR PROMOTE cohort
Source: PLoS One. 2024 Mar 11;19(3):e0290285. doi: 10.1371/journal.pone.0290285 (PMC10927155; doi:10.1371/journal.pone.0290285)
Supplement: Attachment — Submitted filename: Review_UnintendedPregnancyPROMOTE_btb.docx [file pone.0290285.s001.docx]

**Review: Unintended pregnancy and contraception use among African women living with HIV: baseline analysis of the multi-country US PEPFAR PROMOTE cohort.**

Abstract –

- Please separate the methods and results sections
- Methods: Cohort “at” enrolment?
- It’s not clear in the abstract if the Poisson regression was conducted with the enrolment cohort, or later after an intervention with this cohort. Perhaps clarify that you used it on the enrolment data only, and what period of recall was included (if relevant)
- Your methods need to reflect that enrolment was of women with >5 years on ART
- Findings: Close brackets after p=0.054. (and use ‘=’ and not ‘<’ since you’re being precise with the 0.054)
- Conclusions: the final sentence is grammatically incorrect – please separate the final segment of the sentence into its own sentence – looks like it’s meant to be a recommendation statement.

Introduction –

- “S&RH” or SRH? Please confirm norms for acronym in the literature
- Half of WLHIV pregnancies or half of all pregnancies are unintended?
- Read carefully for grammatical correctness.
- In the paragraph starting with ‘unfortunately’, I think the language needs to be revised. The clear implication as it’s currently stated is that we should reduce the number of babies that WLHIV should have – this is very politically incorrect. I suggest restructuring the sentence so the second reason comes first (maternal health), and then reframe it that even in a context of highly effective PMTCT, the more babies that are born to WLHIV will equate to a higher absolute number of vertical transmissions.

Methods –

- Study procedures – you need to mention the >5 years on ART. This is mentioned at the end of the intro, but should be clear here that it was an eligibility criterium
- Analysis – correct ‘a prior’ to ‘a priori’
- (as in the abstract comments) It’s not clear if the Poisson regression was conducted with the enrolment cohort, or later after an intervention with this cohort. Perhaps clarify that you used it on the enrolment data only, and what period of recall was included (if relevant). As most readers will be familiar with Poisson regression only being used for analysis over time, this could be confusing as it’s currently written.

Results -

- You present median duration as 5 years, but the final introductory sentence says “on ART for more than 5 years” and your methods doesn’t indicate anything about ART duration as enrolment criteria. Suggest you clarify across all sections.
- Where describing VL, please add percentages
- How do you define “pregnancy potential” and “a biological potential to be come pregnant”? Usually would be ‘of reproductive age’, but your language choice implies additional criteria – please ensure that definition is included in your methods if so. And if not, consider changing wording
- Marriage/Sex/wanting children – please remove the words “while” and “only” – this implies the addition of your personal opinion, rather than purely reporting the findings. Note – this is a very interesting finding, and perhaps worth a mention in the discussion. Quantitative findings give the “what” and it would be helpful if qualitative research could assist with answering the “why” in future studies.

Discussion -

- P15 – it’s not clear *why* your cohort has such a small age range (28-35) – if this is a function of them being a continuing cohort from a previous study, it would be helpful to describe that in the methods, and as a limitation.
- As a limitation – the limited age range leaves out the younger ages who may have much higher rates of unintended pregnancy, and the older ages, who may have lower rates of unintended pregnancy. Therefore your study has selection bias and can’t be generalized to all women of reproductive age in these countries.

Conclusion –

- Consider how the comments on choice/options could be interpreted by those in severely resource-constrained governments, and what message you’re trying to leave with the reader – in most of these settings, it’s not a realistic expectation that all women “are availed the whole spectrum of effective contraception options” – national health systems can’t afford this. But they should provide options for each of the main categories of contraceptive methods at least at the district hospital level (since it won’t be possible to always decentralize further).
- Variation in choice may also be influenced a lot by the health system – you can only access what’s been ordered and what’s in stock, whether it’s your preferred method or not.
